# Diurnal variability of fine-particulate pollution concentrations: data from 14 low- and middle-income countries

**DOI:** 10.5588/ijtld.20.0704

**Published:** 2021-03-01

**Authors:** R. Dobson, K. Siddiqi, T. Ferdous, R. Huque, M. Lesosky, J. Balmes, S. Semple

**Affiliations:** 1 Institute for Social Marketing and Health, University of Stirling, Stirling, Scotland; 2 Department of Health Sciences, University of York, York, UK; 3 Advancement through Research and Knowledge Foundation Bangladesh, Dhaka, Bangladesh; 4 Division of Epidemiology & Biostatistics, School of Public Health & Family Medicine, University of Cape Town, Cape Town, South Africa; 5 Department of Medicine, University of California, San Francisco, CA; 6 School of Public Health, University of California, Berkeley, CA, USA

**Keywords:** air pollution, lung disease, air quality, LMIC

## Abstract

**BACKGROUND:**

Scientific understanding of indoor air pollution is predominately based on research carried out in cities in high-income countries (HICs). Less is known about how pollutant concentrations change over the course of a typical day in cities in low- and middle-income countries (LMICs).

**OBJECTIVE:**

To understand how concentrations of fine particulate matter smaller than 2.5 microns in diameter (PM_2.5_) change over the course of the day outdoors (across a range of countries) and indoors (using measurements from Dhaka, Bangladesh).

**DESIGN:**

Data on PM_2.5_ concentrations were gathered from 779 households in Dhaka as part of the MCLASS II (Muslim Communities Learning About Second-hand Smoke in Bangladesh) project, and compared to outdoor PM_2.5_ concentrations to determine the temporal variation in exposure to air pollution. Hourly PM_2.5_ data from 23 cities in 14 LMICs, as well as London (UK), Paris (France) and New York (NY, USA), were extracted from publicly available sources for comparison.

**RESULTS:**

PM_2.5_ in homes in Dhaka demonstrated a similar temporal pattern to outdoor measurements, with greater concentrations at night than in the afternoon. This pattern was also evident in 19 of 23 LMIC cities.

**CONCLUSION:**

PM_2.5_ concentrations are greater at night than during the afternoon in homes in Dhaka. Diurnal variations in PM_2.5_ in LMICs is substantial and greater than in London, Paris or New York. This has implications for public health community approaches to health effects of air pollution in LMICs.

## Abstract (FR)

**CONTEXTE:**

La compréhension scientifique de la pollution de l’air intérieur est avant tout basée sur les recherches effectuées dans les villes des pays à revenu élevé (HIC). On en sait moins sur la façon dont les concentrations de polluants évoluent lors d’une journée dans les villes des pays à revenu faible et modéré (LMIC).

**OBJECTIF:**

Comprendre comment les concentrations de particules de diamètre <2.5 μm (PM_2.5_) évoluent dans la journée à l’extérieur (dans un ensemble de pays) et à l’intérieur (grâce à des mesures effectuées à Dhaka, Bangladesh).

**SCHÉMA:**

Des données relatives aux concentrations ont été recueillies à partir de 779 foyers de Dhaka dans le cadre du projet MCLASS II (Muslim Communities Learning About Second-hand Smoke in Bangladesh) et comparées aux concentrations extérieures de PM_2.5_ afin de déterminer la variation temporelle de l’exposition à la pollution aérienne. Des données d’évolution horaires de PM_2.5_ dans trois villes de 14 LMIC, ainsi que de Londres (Royaume Uni), de Paris (France) et de New York (NY, USA), ont été extraites de sources disponibles au public à des fins de comparaison.

**RÉSULTATS:**

Les PM_2.5_ dans les foyers de Dhaka ont mis en évidence un profil temporel similaire aux mesures extérieures, avec des concentrations plus élevées la nuit par rapport à l’après-midi. Ce profil a été retrouvé dans 19 des 23 villes des LMIC.

**CONCLUSION:**

Les concentrations de PM_2.5_ sont plus élevées la nuit que l’après-midi dans les foyers de Dhaka, bien que nos résultats soient limités par la disponibilité d’une seule référence extérieure dans la ville. La variation diurne des PM_2.5_ dans les LMIC est substantiel et plus élevée qu’ à Londres, Paris ou New York. Ceci a des implications en termes d’approches de santé publique des effets sanitaires de la pollution aérienne dans les LMIC.

Air pollution is a serious cause of ill health worldwide, but particularly in low- and middle-income countries (LMICs).^1^ Health messages on air pollution, such as those promulgated by the WHO^2^ and the US Environmental Protection Agency,^3^ have historically been based around 24 h average exposure to levels of pollutants. Much of the evidence which has underpinned these recommendations is based on empirical studies conducted in industrialised cities in Europe and North America.

Temporal data from European and North American cities typically show lower levels of outdoor air pollution than in LMICs.^4^ In these cities in high-income countries (HICs), higher levels of outdoor air pollution are often related to transportation activity, with morning and evening rush hours causing peaks in associated pollutants such as fine particulate matter smaller than 2.5 microns in diameter (PM_2.5_).^5^,^6^ When developing health guidance, these assumptions are considered to hold true for all urban settings.

In many countries, public health authorities provide forecasts, advice and warnings about outdoor air pollution, recommending (for instance) avoiding activities such as physical exercise when air pollution is at its highest.^7^,^8^ This has led to recommendations in the scientific literature^9^ and the media^10^ to avoid exercising during times perceived to have high pollution, such as during rush hours. This may not effectively lower exposure to air pollution, as the concentration of pollution is affected both by production and by dispersal, which may differ in LMICs.

Dispersal of particulate air pollution is strongly influenced by conditions in the atmospheric planetary boundary layer (PBL). The PBL is the lowest layer of the troposphere, where conditions are strongly affected by the Earth’s surface.^11^ Particulate matter (PM) mixes within the PBL, dispersing both vertically and horizontally. The height of the PBL changes with topography and with prevailing conditions, particularly solar radiation and wind. It can vary from as low as 50–100 m (during still, cool periods such as at night) to as high as 5 km (in very hot conditions such as over a desert). These changes occur both seasonally and during the course of the day.

As the height of the PBL changes, the concentration of PM within the atmosphere will change, as it is dispersed within larger or smaller volumes of air. This can be conceptualised by thinking of smoking within a room with a ceiling of variable height: as the ceiling lowers the concentrations of secondhand tobacco smoke will increase due to the reduced room size and volume. The change in altitude of the PBL could lead to counter-intuitive effects—in locations where large diurnal variations in PBL height occur, outdoor concentrations of PM may be lower during the day (despite industrial or transport activity creating PM) and higher as night progresses (as PBL height falls, effectively compressing PM in the atmosphere into a smaller volume).^12^,^13^

Neutral flow occurs in the absence of surface heating (by the sun), resulting in a stable PBL, often associated with night or strong cloud cover. A convective boundary layer occurs when surface heating creates thermals and plumes, causing air to rise up to the top of the boundary layer and creating convective conditions, resulting in a ‘‘mixed layer’’ in the outer region of the PBL. Over time, a highly convective boundary layer effectively erodes the stable layer above it (a ‘‘capping inversion’’), causing that layer to gain in height (to as much as 5 km under particularly convective conditions, such as over a desert in mid-summer, but generally below 2–3 km). By contrast, a stable PBL is not so well defined and has a height above the surface rarely greater than several hundred metres (and, under particularly stable conditions with clear skies and only light winds, the PBL could be as low as 50–100 m).^14^

It has been generally accepted that this effect can result in greater concentrations of pollutants as PBL height falls. However, as reliable and consistent data on PM_2.5_ have not historically been available in LMICs, it had not been possible previously to observe the magnitude of this effect without complex and expensive programmes of measurement. As interest in outdoor air pollution in these settings has grown, however, more data have become available. The United States has sited a number of high-quality PM_2.5_ monitors within range of its embassies and consulates in LMICs. These monitors provide continuous hourly data on PM_2.5_ concentrations at each site.

The recent availability of low-cost particle counting monitors has also made it possible to assess concentrations of PM more widely, both indoors and outdoors. As part of the MCLASS II (Muslim Communities Learning About Second-hand Smoke in Bangladesh) study,^15^ a trial of a novel smoke-free homes intervention, 24 h measurements of indoor PM were made in 1,801 homes around Dhaka, Bangladesh, using one such monitor, the Dylos DC1700 particle counter (Dylos Corp, Riverside, CA, USA). These data included 779 homes where smoking was not reported to take place. To the authors’ knowledge, this represents the largest existing set of such measurements in homes in South Asia. As such, this data set provides a valuable opportunity to investigate the impact of diurnal change in PM_2.5_ concentration on concentrations indoors, and consequently on personal exposure to PM_2.5_.

To this end, we present data from the US Embassy & Consulate PM_2.5_ monitoring stations in LMIC settings across four continents to analyse diurnal change in PM_2.5_ concentration. We further examine this effect through 24-h measurements made in 779 homes in Dhaka, Bangladesh, over the course of 5 months in 2018.

## METHODS

### Data from homes in Bangladesh

Data from 1801 households in Dhaka, Bangladesh, were collected between April and August 2018 as part of the MCLASS II project—a research study evaluating a programme to encourage smoke-free homes using a faith-based intervention.^15^ Around 40 households (with at least one smoker and one non-smoker living at home) were recruited from the catchment area of each of 45 mosques in Mirpur, Dhaka. To approach and screen the mosques, we collected a list of registered mosques within our study area from the Islamic Foundation Bangladesh, Dhaka, Bangladesh (an autonomous body under the Ministry of Religious Affairs). Following the list, mosques were selected by agreement to participate in the MCLASS II intervention study; leadership of a non-smoking *imam* or *khatib* (who could deliver intervention components); and distance of at least 500 m from another participating mosque. The location of each mosque within Dhaka is shown in Supplementary Figure S1.

Air quality monitoring was conducted using Dylos DC1700 particle counters (Dylos Corporation). Monitors were installed in the main living area of the home (excluding the kitchen and window side) by trained fieldworkers and left to function for 24 h. Dylos particle number concentrations were converted to estimated PM_2.5_ mass concentrations using a previously derived conversion equation based on comparisons with a TSI SidePak™ (TSI Incorporated, Shoreview, MN, USA) instrument.^16^

As part of the larger MCLASS II project (an intervention study intended to reduce smoking indoors among participants in Dhaka), participants were asked about potential sources of PM_2.5_ in the home. Homes where biomass fuels were used indoors were ineligible to take part in the overall study. Only data from homes which were reported by the participant to be smoke-free—where no-one smoked indoors—were included in this analysis to minimise the potential confounding factor of tobacco smoke leading to unrelated high concentrations of PM_2.5_ in the home; 779 households (43% of the total) met this criterion and were included. Mean concentrations by hour were calculated for each home; arithmetic means were then calculated across all 779 homes for each hour.

Participants provided informed consent as part of the MCLASS II project. MCLASS II has been granted ethical approval by the National Research Ethics Committee of the Bangladesh Medical Research Council, Dhaka, Bangladesh (Ref: BMBC/NREC/ 2016–2019/358) and the Health Sciences Research Governance Committee at the University of York, York, UK (no reference number, approval date 8 August 2017).

### Outdoor air pollution data

Mean PM_2.5_ concentration data were acquired from 25 US embassy or consulate PM_2.5_ monitoring stations operating in 16 LMICs in 2018 across Asia, Africa, South America and Europe. These data are freely available on the internet.^17^ The location of the monitoring station in Dhaka is shown in Supplementary Figure S1. There were no reliable air pollution monitoring data available from government sources in these countries. Raw concentration data were used rather than the provided NowCast (Economic Alchemy, New York, NY, USA) data, as this method involves a weighted average of concentrations over the last 3–12 h, conflicting with the aim of this study to examine concentrations at each hour.^18^

Data were cleaned by removing results listed as ‘‘-999’’ and removing data listed as ‘‘Missing’’ or ‘‘Invalid’’ by the embassy’s quality control, in line with recommended handling of this data set. Data integrity was determined for each monitor by calculating the percentage of hours with data compared to the total number of hours in the year.

To allow comparisons by broad geographical region, including climate and weather, and to ensure that data were available for each month, data from the US embassy monitoring stations were grouped according to UN M.49 geoscheme sub-regions (an international standard means of grouping nations into geographic regions).^19^ Arithmetic mean concentrations were calculated for each hour of the day for each site and region to observe seasonal and diurnal changes.

To provide comparative data on diurnal variations in PM_2.5_ concentrations from urban centres in HICs, hourly outdoor measurements for London (UK), New York City (NY, USA) and Paris (France) were extracted from the Automatic Urban and Rural Network (London, UK), the UK’s automatic PM monitoring network, the New York State Department of Environmental Conservation Database (New York, NY, USA) and the European Environment Agency AirBase (Copenhagen, Denmark), respectively. Means were taken for each of 12 monitors in London, 11 in New York and 6 in Paris for each hour in 2018; mean concentrations and standard deviations were then calculated for each hour of the day.

### Statistical analysis

Descriptive statistics were calculated for both outdoor and indoor data sets. Linear mixed-effects models were fit to indoor air pollution data to estimate associations with time of day, adjusting for outdoor air pollution, and calendar month, and fitting a random effect for household ID nested within calendar month. Hour in day associations were fit using a four-level discrete variable with 6 h time periods: 02:00–08:00, 08:00–14:00, 14:00–20:00 and 20:00–02:00. Indoor and outdoor air pollution levels were entered as natural log-transformed variables, but interpreted on original scales. Statistical analysis was conducted using IBM SPSS Statistics v23 (IBM Corp, Armonk, NY, USA), R (R Computing, Vienna, Austria) and Python 2.7 (Python Software, Wilmington, DE, USA).

## RESULTS

### Hourly variations in indoor and outdoor air pollution in Dhaka

The descriptive statistics for the data from 779 smoke-free homes drawn from the MCLASS II study^15^ are shown in Table 1. The median 24-h mean PM_2.5_ concentration in homes in Dhaka was 27.2 μg/m^3^—exceeding the WHO’s guidance limit of 25 μg/m^3^ over 24 h.

**Table 1:** Descriptive statistics for MCLASS II smoke-free homes

*n*	Duration (minutes) Median [IQR]	24 h mean PM_2.5_ concentration (μg/m^3^) Median	24 h PM_2.5_ concentrations (μg/m^3^) Mean [IQR]	Lowest 24 h mean PM_2.5_ concentration (μg/m^3^)	Highest 24 h mean PM_2.5_ concentration (μg/m^3^)
779	1440 [1440–1440]	27.2	24.5 (19.4–43.9)	6.3	290.5

MCLASS II = Muslim Communities Learning About Second-hand Smoke in Bangladesh; IQR = interquartile range; PM_2.5_ = particulate matter with diameter below 2.5 μm.

**Figure 1.**
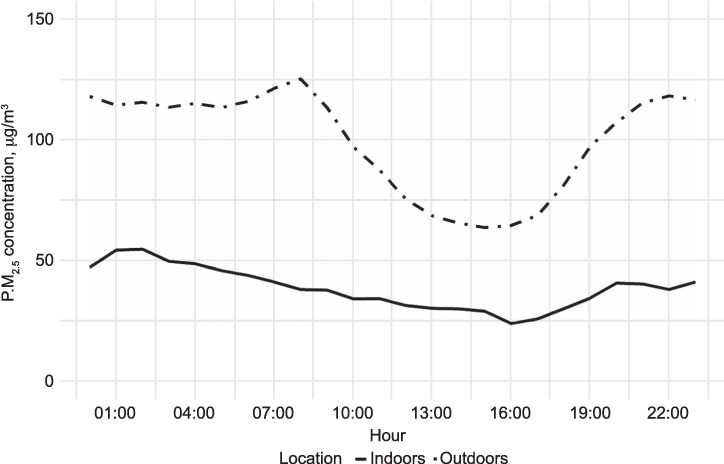
PM_2.5_ measurements by hour, US embassy, Dhaka, 2018, compared to indoor PM_2.5_ measurements by hour in MCLASS II smoke-free homes. Note that each hour represents the mean of all data collected in the preceding 60 min. PM_2.5_ = particulate matter smaller than 2.5 μm in diameter; MCLASS II = Muslim Communities Learning About Second-hand Smoke in Bangladesh.

**Figure 2.**
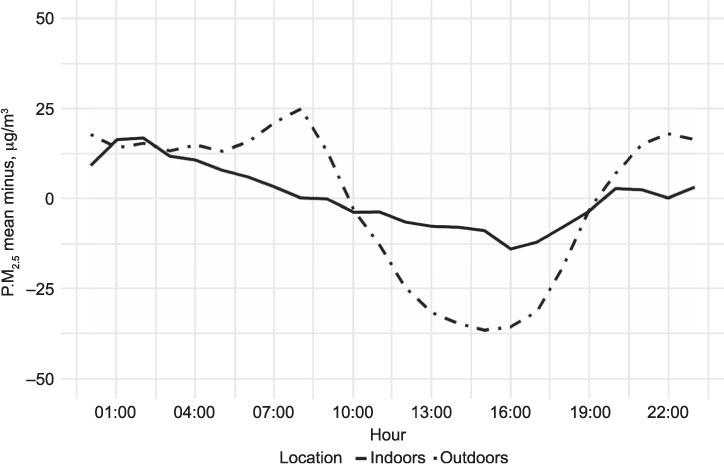
Difference between 24-h PM_2.5_ mean and hourly mean for outdoor US embassy, Dhaka, and for indoor PM_2.5_ MCLASS II smoke-free homes measurements. Note that each hour represents the mean of all data collected in the preceding 60 min. PM_2.5_ = particulate matter smaller than 2.5 μm in diameter; MCLASS II = Muslim Communities Learning About Second-hand Smoke in Bangladesh.

These data were compared to outdoor PM_2.5_ data from Dhaka’s US embassy reference PM_2.5_ monitor, which showed a mean annual PM_2.5_ concentration of 100 μg/m^3^ (Table 2), greatly in excess of the WHO guideline limit. Hourly mean PM_2.5_ concentrations for both outdoor and indoor data were closely related, showing declines in the afternoon and rising overnight (Figure 1). This suggests that changes in outdoor air pollution (driven by PBL height changes) are reflected in changes in household indoor air PM_2.5_ concentrations. The estimated association between indoor and outdoor air pollution is that a 1% increase in outdoor air pollution corresponds to a 0.3% (95% confidence interval [CI] 0.29–0.33) increase in indoor air pollution, adjusted for season and time of day. Similarly, time of day remained significantly associated with changes in indoor air pollution after adjusting for outdoor levels and season (*P* < 0.001). Compared to late afternoon (14:00–20:00) all other periods were modelled to have higher levels of indoor air pollution on average, with an estimated increase of 1.20 μg/m^3^ (95% CI 1.17–1.24) during the evening/night (20:00–02:00), 1.22 μg/m^3^ (95% CI 1.19–1.26) during the late night (02:00–08:00) and 1.13 μg/m^3^ (95% CI 1.10–1.16) during the day (08:00–14:00). In general, indoor air pollution was lower (Figure 1) and did not vary as greatly over the course of the day as outdoor air pollution (Figure 2).

**Table 2:** Outdoor PM_2.5_ concentrations by monitor location

Site	Region	PM_2.5_ concentration, μg/m^3^ Mean ± SD	PM_2.5_ concentration, μg/m^3^ Median [IQR]	Difference between highest hourly and lowest hourly average (μg/m^3^)
Addis Ababa, Ethiopia	Eastern Africa	37 ± 28	26 [20–46]	43
Kampala, Uganda	Eastern Africa	59 ± 33	40 [35–75]	44
Beijing, China	Eastern Asia	51 ± 49	55 [14–69]	10
Chengdu, China	Eastern Asia	51 ± 31	35 [29–64]	12
Guangzhou, China	Eastern Asia	32 ± 22	23 [18–41]	5
Shanghai, China	Eastern Asia	36 ± 30	22 [19–41]	3
Shenyang, China	Eastern Asia	42 ± 35	39 [18–57]	21
Ulaanbaatar, Mongolia	Eastern Asia	62 ± 106	52 [9–61]	92
Bogota, Colombia	South America	14 ± 10	14 [6–20]	15
Lima, Peru	South America	32 ± 17	17 [21–38]	15
Hanoi, Viet Nam	Southeastern Asia	41 ± 32	32 [19–51]	12
Ho Chi Minh City, Viet Nam	Southeastern Asia	26 ± 18	18 [15–33]	13
Jakarta South, Indonesia	Southeastern Asia	45 ± 26	34 [26–60]	22
Chennai, India	Southern Asia	30 ± 27	23 [14–37]	16
Colombo, Sri Lanka	Southern Asia	32 ± 19	21 [19–40]	12
Dhaka, Bangladesh	Southern Asia	100 ± 84	109 [37–146]	62
Hyderabad, India	Southern Asia	59 ± 33	42 [35–77]	33
Kathmandu, Nepal	Southern Asia	91 ± 86	88 [34–122]	55
Kolkata, India	Southern Asia	75 ± 52	60 [39–99]	48
Mumbai, India	Southern Asia	106 ± 96	99 [43–142]	38
New Delhi, India	Southern Asia	58 ± 46	58 [23–81]	67
Pristina, Kosova	Southern Europe	30 ± 41	18 [11–29]	26
Sarajevo, Bosnia and Herzegovina	Southern Europe	39 ± 59	22 [14–36]	16
London, UK	London	11 ± 2	11 [10–12]	3
New York, NY, USA	New York	7 ± 1	8 [7 – 8]	2
Paris, France	Paris	14 ± 2	14 [13 – 16]	4

PM_2.5_ = particulate matter smaller than 2.5 μm in diameter; SD = standard deviation; IQR = interquartile range.

### Outdoor air pollution by region

Monitors in Astana, Kazakhstan, and Tashkent, Uzbekistan, reported no data before May or June 2018. These monitors were therefore excluded from the analysis, leaving 23 monitoring sites across 14 countries in six regions to be included—Southern Europe (one monitor in Kosovo and one in Bosnia & Herzegovina), Eastern Asia (five in China and one in Mongolia), Eastern Africa (one in Uganda and one in Ethiopia), South America (one in Peru, one in Colombia), Southern Asia (five in India, one in Sri Lanka, one in Bangladesh and one in Nepal) and Southeastern Asia (two in Viet Nam and one in Indonesia). Annual PM_2.5_ data for each site is given in Table 2.

### Hourly variations in outdoor air pollution

**Figure 3.**
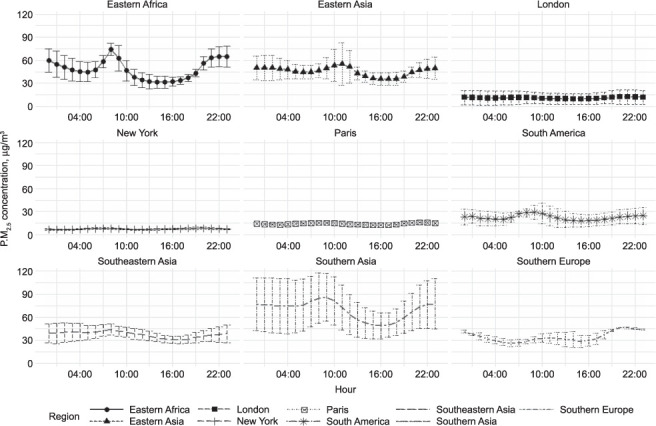
Regional US embassy and consulate PM_2.5_ measurements by hour, 2018, including values for London, New York and Paris as comparators. Error bars represent standard deviations. PM_2.5_ = particulate matter smaller than 2.5 μm in diameter.

**Figure 4.**
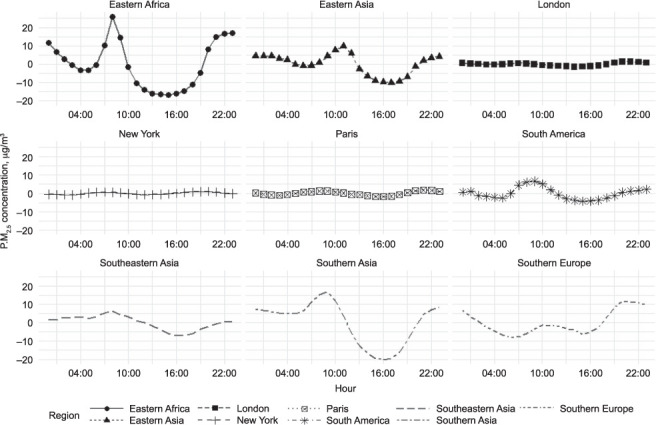
Hourly mean difference from overall annual mean, regional US embassy PM_2.5_ measurements. Data for London, New York and Paris are provided as a comparator. PM_2.5_ = particulate matter smaller than 2.5 μm in diameter.

Across all regions, daily outdoor PM_2.5_ concentrations peak in the morning (between 06:00 and 11:00), then decline until the late afternoon, when they begin to rise once again (Figure 3). All regions saw a large increase in mean hourly concentrations compared to mean daily concentrations in the morning, sometime between 06:00 and 11:00, likely reflecting morning commuter traffic across the city (Figure 4). This effect could be stark—in Eastern Africa, for example, the mean concentration in the mid-afternoon was around half the mean during the mid-morning peak.

**Figure 5.**
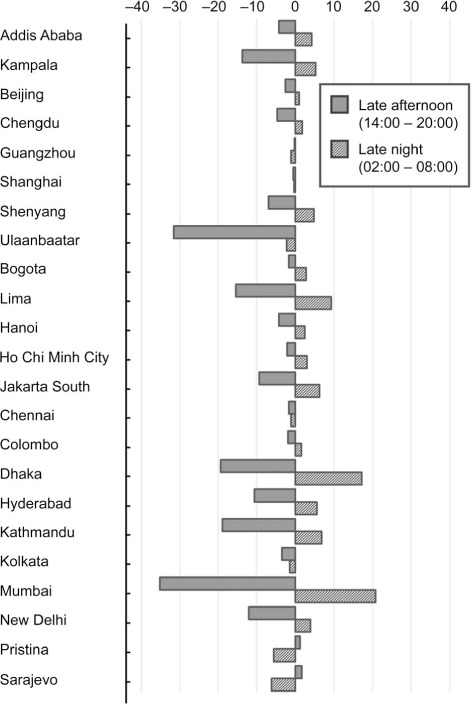
Difference between mean annual outdoor PM_2.5_ concentration in the late afternoon (14:00–20:00) and at night (02:00–08:00) by LMIC city, 2018. PM_2.5_ = particulate matter smaller than 2.5 μm in diameter; LMIC = low- and middle-income country.

To estimate the extent of the difference at times of the day when activity patterns are likely to differ, the mean PM_2.5_ concentrations recorded by each monitor in the late afternoon (14:00–20:00) and night (02:00–08:00) were compared (Figure 5). Mean overnight concentrations were higher than afternoon concentrations in 19 of the 23 cities. Overnight concentrations were a median 26% higher than the afternoon concentrations. The median difference between the highest average hourly and the lowest average hourly across all 23 sites was 26 μg/m^3^; nine of the 23 cities showed swings of >40 μg/m^3^.

By way of comparison, the hourly average PM_2.5_ concentrations in HICs demonstrated very little variation across 2018. The overall average in London during this period was 11.3 μg/m^3^, with the range between the highest hourly average and the lowest hourly average being 2.8 μg/m^3^. New York and Paris showed similarly small changes in their relatively low levels of ambient PM_2.5_.

## DISCUSSION

Environmental exposure science has been dominated since its inception by researchers from HICs, particularly Europe and the United States. Our results show that average hourly PM_2.5_ concentrations in London during 2018 was within ±2 μg/m^3^. By contrast, PM_2.5_ concentrations in a range of cities in Southern Asia varied by ±15 μg/m^3^.

Diurnal variation in outdoor air PM_2.5_ concentrations is not driven solely by industrial activities or by transportation in the LMICs studied, but may also be affected by changes in atmospheric mixing height. This is particularly evident in southern Asia (including cities in India and Bangladesh) and east Africa, and may contrast with the expectations of scientists who have conducted exposure monitoring studies in areas which do not experience this stark effect. Exposure scientists in the global north may not take the magnitude of diurnal variation in LMICs into account when designing research programmes.

In homes in Dhaka, diurnal variation in PM_2.5_ concentrations is related closely to changes in outdoor air, with changes occurring at the same time as changes in PBL height would be expected. This has implications for studies of indoor air pollution and personal exposure to air pollution, and may have serious implications for the health of people living in these environments. Messaging around the health impacts of physical activity, for example, should take into account the timing of that activity— someone in Dhaka exercising outdoors at 17:00 will, on average, be exposed to concentrations of PM_2.5_ of 85 μg/m^3^; the same individual performing exercise at 07:00 will, on average, inhale concentrations of 141 μg/m^3^,a 66% increase. By comparison, a jogger in London exercising at these two different times will typically inhale concentrations of 16.4 μg/m^3^ (17:00) and 16.9 μg/m^3^ (07:00)—a difference unlikely to have any physiological effect.

Epidemiological data show a strong association between increases in outdoor PM_2.5_ concentrations and adverse health effects. The most recent meta-analysis suggests an approximate 1% increase in mortality for every 10 μg/m^3^ PM_2.5_, with a similar association for hospital admissions.^20^ These acute-effect studies are often based on modelled 24 or 72 h average PM_2.5_ concentrations.^21^–^23^ Increases over shorter periods may generate similar changes in mortality and morbidity. He et al. demonstrated that 60 min of exposure to high concentrations of PM_2.5_ can lead to arrhythmia,^24^ while Garza et al. showed that participants exposed to secondhand smoke for 6 h could experience lowered heart rate variability.^25^ Returning to the temporal variation in concentrations in Dhaka and the 56 μg/m^3^ difference in PM_2.5_ exposures that may occur between 07:00 and 17:00, it seems plausible that this difference could generate differing effects on public health.

It should be noted that a morning peak visible in outdoor air pollution data in Dhaka was not visible in indoor data. This could hypothetically be explained by a localised increase in pollution during this time, affecting the US embassy monitor within the Dhaka central business district but not homes outside of the city centre.

There are clear and important implications both for air pollution health studies in these countries, and perhaps more immediately, for health advice and health service provision for acute cardio-respiratory illness-related to high exposure to pollutants. For example, exacerbations of asthma and chronic obstructive pulmonary disease (COPD) may peak at very different times in Eastern Africa or South-Eastern Asia compared to the United States or Europe, and such knowledge may enable better targeting of resources and public health messaging.

### Limitations

Outdoor PM_2.5_ data in LMICs were derived solely from US State Department reference monitors, with one located in each city. This may not accurately report citywide ambient PM_2.5_ concentrations, which could differ by location (due to industrial or agricultural pollution, for example). The location of US embassies and consulates (and, consequently, their associated PM_2.5_ monitors) may not reflect settlement patterns in each city—for example, in Dhaka the US embassy is sited within the central business district of the city, around 3–4 km from Mirpur where much of the MCLASS II in-home monitoring took place. The early morning peak visible in most regions was not reflected in indoor data from Dhaka. Embassy monitors may be more centrally located than homes, experiencing a greater effect from the morning rush hour than sites in other areas and accounting for this peak.

Several US embassy and consulate sites were missing data for one or more months, which (given the highly seasonal nature of air pollution and of the PBL in many settings) could affect the accuracy of these results. However, the large amount of data available and the consistent results seen in this analysis suggest that missing data are unlikely to have had a significant effect on overall results.

All households recruited in the MCLASS II project had at least one smoker present as an inclusion criteria. These smokers may have exaggerated their efforts to smoke outside the home, so the homes included in this analysis may not truly have been ‘‘smoke-free’’. However, a close relationship between changes in outdoor and indoor air pollution was observed suggesting that ambient air pollution was the primary driver of air quality in these homes.

## CONCLUSIONS

Studies of the effect of air pollution on health in LMIC cities with high levels of PM_2.5_ should take into account the extent and patterns of diurnal variations and consider the possibility that this may be much greater than and different from that seen in HICs. For health services, particularly in relation to cardio-respiratory health, the implications of these temporal changes in pollutant concentrations over the course of the day may be substantial.

Designers of personal exposure monitoring studies should take into account the effect observed in this study—that meteorological features differ by country and region—and take care to avoid importing assumptions from North American or European settings to LMICs, particularly those in southern and eastern Asia and sub-Saharan Africa.
